# Case Report: Neuromyelitis optica spectrum disorder associated with gastric cardia and pancreatic cancers: clinical features and oncological implications

**DOI:** 10.3389/fonc.2026.1799562

**Published:** 2026-06-03

**Authors:** Xiaoshan Wei, Kun Yuan, Wei Sang, Hongwei Zhang, Yidi Qin

**Affiliations:** 1 Department of Neurology, Shijiazhuang Traditional Chinese Medicine Hospital, Shijiazhuang, Hebei, China; 2 Department of Acupuncture, Shijiazhuang Traditional Chinese Medicine Hospital, Shijiazhuang, Hebei, China; 3 Department of Radiology, Shijiazhuang Traditional Chinese Medicine Hospital, Shijiazhuang, Hebei, China

**Keywords:** AQP4, case report, gastric cardia cancer, inflammatory demyelinating pseudotumor, neuromyelitis optica spectrum disorder (NMOSD), pancreatic cancer, paraneoplastic NMOSD (PNNMOSD)

## Abstract

This article reports two elderly patients with Neuromyelitis Optica Spectrum Disorder (NMOSD) associated with gastric cardia cancer and pancreatic cancer, respectively each presenting distinctive clinical manifestations and diagnostic challenges. Case 1 involved a 69-year-old male who developed stroke-like symptoms after surgery for gastric cardia cancer, which later progressed to optic neuritis, confirmed by positive aquaporin-4 (AQP4) antibodies in both serum and cerebrospinal fluid. Case 2 was a 64-year-old female whose initial presentation included optic neuritis and myelitis. Early cervical spinal MRI revealed a focal space-occupying lesion, and AQP4 antibodies were negative, complicating the diagnosis. However, the diagnosis was ultimately established based on characteristic clinical symptoms, a marked response to corticosteroid therapy, and the appearance of longitudinally extensive transverse myelitis (LETM) on imaging during relapse. This report describes the dynamic imaging evolution of spinal cord inflammatory demyelinating pseudotumor in NMOSD. It also expands the spectrum of tumors associated with NMOSD to include gastric cardia cancer and pancreatic cancer. It emphasizes that in elderly patients or those with atypical NMOSD presentations, potential oncological etiologies should be considered and targeted malignancy evaluation should be performed. Furthermore, a multidisciplinary collaborative approach is essential to develop individualized treatment strategies that simultaneously address both the neoplasm and the immune-mediated demyelinating disorder.

## Introduction

Neuromyelitis Optica Spectrum Disorder (NMOSD) is an immune-mediated inflammatory demyelinating disease of the central nervous system, primarily characterized by targeted attacks on aquaporin-4 (AQP4) water channels located on astrocytic foot processes (1). Its typical clinical manifestations include optic neuritis, acute myelitis, and area postrema syndrome. The disease is also known for its high relapse rate and significant disability burden (2). Recent studies have indicated that some NMOSD cases may be associated with malignant tumors and are thus classified as paraneoplastic NMOSD (PNNMOSD). The pathogenesis is thought to involve molecular mimicry, in which tumor tissues abnormally express AQP4 protein and trigger a cross-reactive immune response that later attacks the central nervous system (3). PNNMOSD represents about 3% to 25% of all NMOSD cases, according to the literature (4). It is most frequently associated with lung cancer and breast cancer, comprising 21.1% and 18.3% of reported cases, respectively (5). Other neoplasms such as ovarian cancer and ovarian teratoma have also been documented in the literature (4–7). Regarding gastrointestinal tumors, NMOSD cases associated with gastric neuroendocrine tumors, intrahepatic cholangiocarcinoma, and rectal cancer have been reported (8–10). This report details two cases of NMOSD associated with gastric cardia cancer and pancreatic cancer: a 69-year-old male with gastric cardia cancer presenting with stroke-like onset that progressed to optic neuritis and longitudinally extensive myelitis, confirmed by strongly positive AQP4-IgG antibodies; and a 64-year-old female with recurrent optic neuritis and transverse myelitis, diagnosed with pancreatic cancer two years later. By presenting two cases featuring distinct clinical presentations of gastric cardia cancer and pancreatic cancer, respectively, we provide novel clinical evidence reinforcing the rationale for comprehensive malignancy screening in elderly NMOSD patients with atypical presentations (11), and underscore the importance of long-term follow-up.

## Case presentation

**Figure 1:**
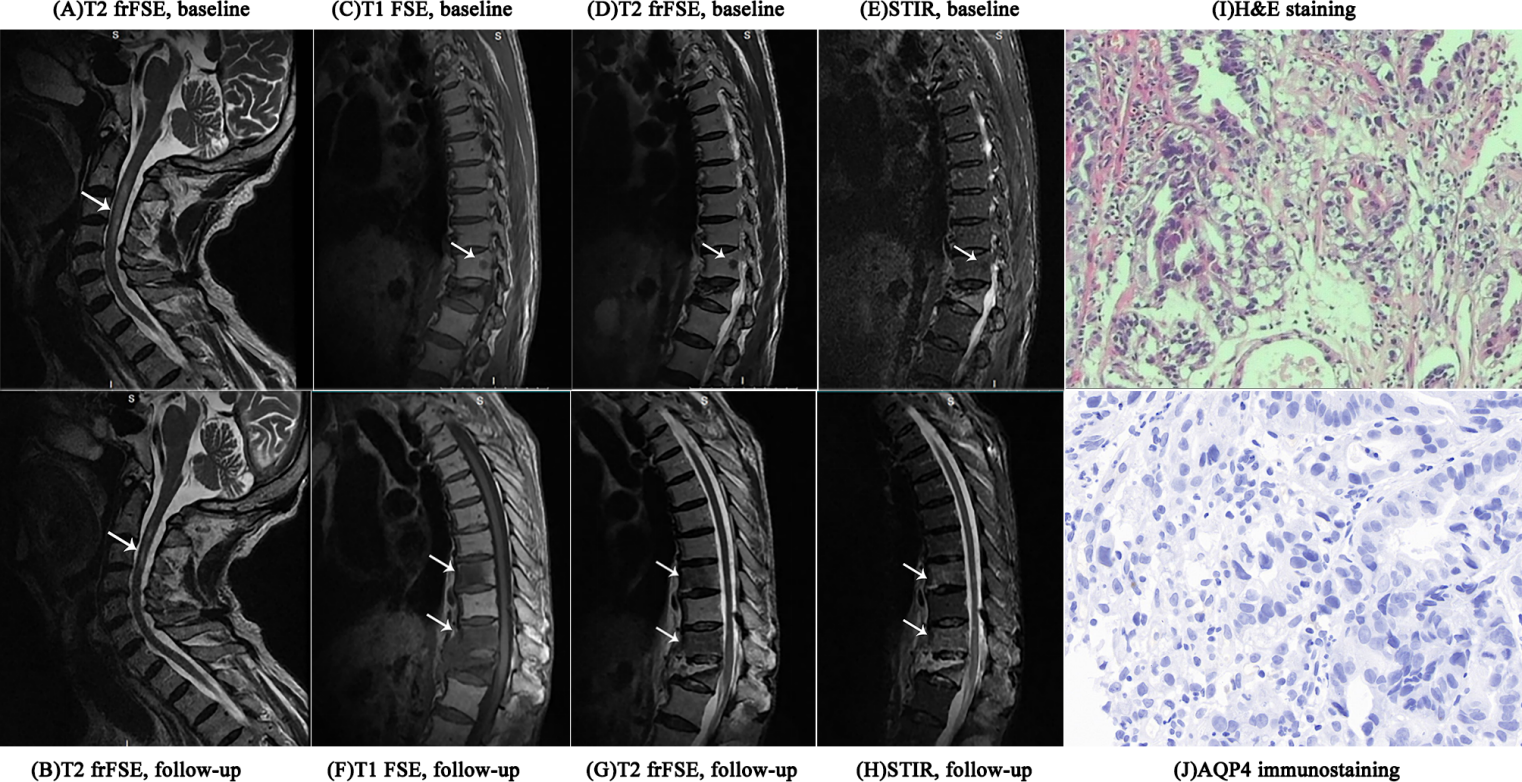
Magnetic resonance imaging (MRI) and pathological findings of Case 1. **(A)** Baseline cervical MRI shows a long T2-hyperintense lesion extending from C2 to C4. **(B)** After one month of treatment, follow-up MRI reveals improvement of the spinal cord signal abnormality at C2–C4. **(C–E)** Baseline thoracic spine MRI: a focal rounded hypointense lesion in the T11 vertebral body on T1-weighted **(C)**, T2-weighted **(D)**, and fat-suppressed sequences **(E)**. **(F–H)** Thoracic spine MRI one month later: extensive involvement of the T9 and T11 vertebral bodies with hypointense signal on T1-weighted **(F)** and T2-weighted **(G)** imaging, and hyperintense signal on fat-suppressed sequences **(H)**. **(I)** Hematoxylin and eosin (H&E) staining of a preoperative gastroscopic biopsy specimen of the gastric cardia cancer. **(J)** Immunohistochemical staining with an aquaporin-4 (AQP4)-specific monoclonal antibody on the same specimen shows negative immunoreactivity.

Case 1. A 69-year-old male presented a sudden onset of right-sided limb weakness. The symptoms had started abruptly 2 days prior, manifesting as impaired movement of the right limbs. The patient had a seven-year history of hypertension and type 2 diabetes mellitus, and underwent radical surgery for gastric cardia cancer 1.5 years ago. Pathology revealed intramucosal adenocarcinoma without lymph node metastasis, staged as pT1aN0M0 (**Figure 1I**). The patient had since undergone regular postoperative follow-up. There was no family history of similar diseases. Physical examination revealed muscle strength of grade IV in the right limbs and left lower limb. Positive bilateral Babinski signs were observed. A head computed tomography (CT) showed a lacunar infarct in the deep left frontal region and leukodystrophy. The initial diagnosis on admission was acute cerebral infarction. However no significant improvement was observed with treatment.

On the 3rd day of hospitalization, the patient developed sudden vision loss in the left eye. Ophthalmology consultation indicated no light perception in the left eye. The pupil diameter of the left eye was about 5mm, and the direct reflection to light disappeared. Optical Coherence Tomography (OCT) showed that the blood flow density in the macular area of both eyes decreased significantly. Orbital CT showed no significant abnormalities. Visual evoked potentials were abnormal on the left. Brain magnetic resonance imaging (MRI) revealed punctate ischemic lesions in the bilateral frontal and parietal lobes. Spine MRI showed a T2 hyperintense intramedullary lesion extending from the C2 to C4 levels (**Figure 1A**). Additionally, a focal rounded lesion was observed in the T11 vertebral body on T1-weighted, T2-weighted, and fat-suppressed sequences (**Figures 1C–E**).

Both serum and cerebrospinal fluid (CSF) tested positive for aquaporin-4 antibodies (AQP4-IgG), whereas test results for antibodies against myelin oligodendrocyte glycoprotein (MOG-IgG) and myelin basic protein (MBP-IgG) were negative, all using a cell-based assay (CBA). The diagnosis was revised to NMOSD. Intravenous methylprednisolone pulse therapy was given. After treatment, visual acuity in the left eye improved to counting fingers at 2 meters, and muscle strength recovered to grade IV+ in the right limbs and left lower limb. Oral methylprednisolone was continued with gradual tapering after discharge.

One month later, the patient presented with significant low back pain. We requested an MRI of the cervical, thoracic, and lumbar spine for the patient. Compared to the prior imaging, the follow-up cervical MRI revealed improvement of the spinal cord signal abnormality at C2-C4 (**Figure 1B**), and the thoracolumbar spine MRI showed T1 and T2 hypointense signals in the T9 and T11 vertebral bodies, suspicious for metastatic lesions (**Figures 1F–H**). The patient was subsequently transferred to a higher-level hospital and lost to follow-up. We evaluated AQP4 expression using immunohistochemistry in gastroscopic biopsy specimens obtained prior to radical gastrectomy for gastric cardia cancer. No positive staining was observed (**Figure 1J**).

Case 2. A 64-year-old female was admitted in December 2021 due to visual impairment. She also experienced numbness and weakness in both upper limbs, along with muscle spasms and pain in the right upper limb. All symptoms had persisted for over six months. She had a history of hypertension spanning several years and underwent a laparoscopic cholecystectomy 8 years prior. On admission, physical examination demonstrated a visual acuity of 0.8 in the right eye and 0.6 in the left eye, with no visual field defects. Decreased pain sensation was noted in both upper limbs, and muscle strength graded IV.

**Figure 2:**
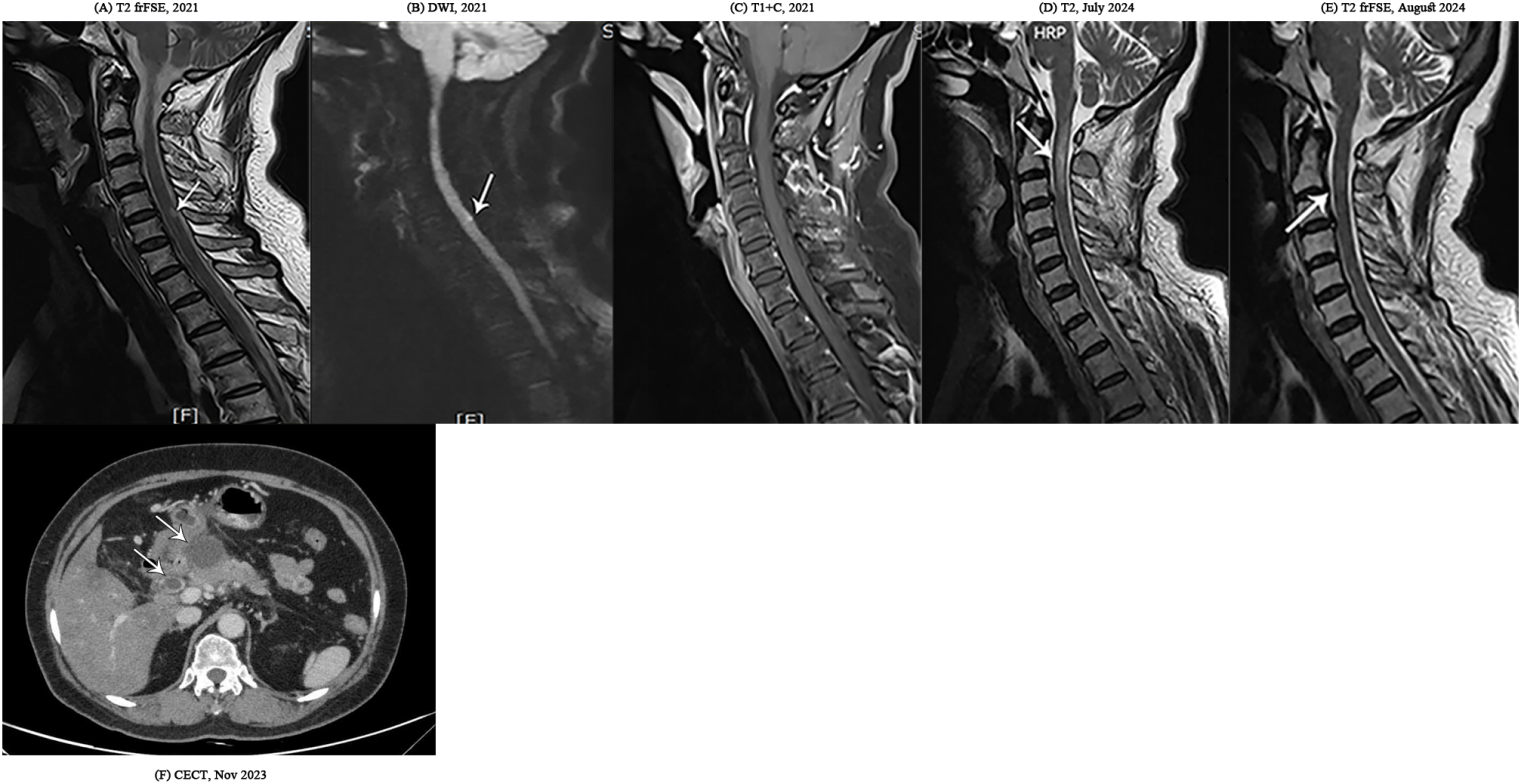
The 2021 MRI showed a nodular intramedullary hyperintensity at the C5–C6 level on T2-weighted **(A)** and DWI images **(B)**, without significant enhancement on the contrast-enhanced image **(C)**. **(D)** The July 2024 MRI revealed a new longsegment T2 hyperintensity within the spinal cord at the C1–C3 levels, concurrent with the resolution of the previously observed space-occupying lesion. **(E)** Follow-up MRI in August 2024 demonstrated marked regression of the C1–C3 long-segment lesion. **(F)** Contrast-enhanced CT on November 27, 2023, revealed multifocal lesions in the pancreatic head, body, and tail, including a cystic lesion in the pancreatic neck, all suspicious for neoplasm. Additionally, diffuse abnormal contrast enhancement was observed in the pancreatic segment of the common bile duct wall, also suggestive of neoplasm and resulting in biliary obstruction.

Cervical MRI showed an intramedullary nodular lesion between the C5 and C6 levels. The lesion exhibited hyperintensity on T2-weighted (**Figure 2A**) and diffusion-weighted imaging (DWI) (**Figure 2B**). No significant enhancement was observed on contrast-enhanced imaging (**Figure 2C**), suggesting an intramedullary space-occupying lesion. CSF biochemistry showed a total protein level of 1.5 g/L. CSF/Serum antibody testing using a cell-based assay (CBA) returned negative results for AQP4-Ab, MOG-Ab, and MBP-Ab. Additional tests, including anti-extractable nuclear antigen (ENA) antibodies, female tumor markers, and rheumatologic panels, were negative.

The patient was initially strongly suspected of having NMOSD and received methylprednisolone pulse therapy. Following treatment, the patient exhibited notable improvement in visual acuity as well as reduction of numbness and weakness in the upper limbs. Additionally, muscle spasms and pain were significantly relieved. Repeat physical examination revealed that muscle strength in both upper limbs had recovered to V^-^ grade. Subsequently, the patient was maintained on long-term oral prednisone tablets at a dose of 5 mg for ongoing maintenance therapy.

On November 16, 2023, the patient presented with painless progressive jaundice. Abdominal MRI (November 17, 2023) revealed a cystic lesion in the pancreatic head, mild dilation of the hilar bile duct and common bile duct, a small amount of perisplenic fluid, and absence of the gallbladder. Contrast-enhanced CT on November 27, 2023 showed lesions in the pancreatic head, body, and tail with a cystic lesion in the pancreatic neck, suspicious for neoplasm; diffuse abnormal contrast enhancement of the pancreatic segment of the common bile duct wall, also suspicious for neoplasm, causing biliary obstruction; heterogeneous decreased liver density suggestive of hepatic injury; and non-visualization of the gallbladder (**Figure 2F**). On November 29, 2023, under general anesthesia, the patient underwent endoscopic ultrasound-guided fine-needle aspiration (EUS-FNA), endoscopic retrograde cholangiopancreatography, endoscopic sphincterotomy, and endoscopic biliary stent placement. Postoperative pathological examination revealed scattered and clustered cancer cells, consistent with a pancreatic ductal origin based on the immunophenotype. Prednisone was discontinued in anticipation of possible surgery, and the patient tried switching to mycophenolate mofetil but stopped due to side effect intolerance. The patient did not undergo surgery. Chemotherapy and immunotherapy were initiated on December 13, 2023, beginning with an intravenous infusion of nab-paclitaxel and gemcitabine hydrochloride; on December 14, she received sintilimab intravenously; and on December 20, she received a second intravenous infusion of nab-paclitaxel and gemcitabine hydrochloride. This regimen was repeated approximately every two weeks for a total of six cycles, with the last cycle completed on April 30, 2024.

In July 2024, the patient experienced a recurrence of symptoms, manifesting as numbness, weakness, and muscle spasms in the upper limbs. Cervical MRI revealed longitudinally extensive T2 hyperintense signals extending from C1 to C3 levels; the previously identified space-occupying lesion between C5 and C6 levels resolved (**Figure 2D**). A repeated course of methylprednisolone pulse therapy significantly improved the symptoms. A follow-up MRI in August 2024 showed a marked reduction in the abnormal signals (**Figure 2E**). The pancreatic cancer continued to progress. According to her husband, "Having experienced the severe adverse effects of chemotherapy, she steadfastly declined further anti-tumor treatment and sought solely to optimize pain management". Later, the patient developed pyloric obstruction, which required a jejunostomy. Ultimately, the patient died from tumor progression in March 2025."

## Discussion

This report describes two cases of NMOSD associated with gastric cardia cancer and pancreatic cancer, respectively. These cases highlight the complex clinical manifestations and the diagnostic and therapeutic challenges associated with the link between NMOSD and underlying malignancies. The distinct clinical courses, treatment responses, and imaging characteristics of these two patients provide valuable clinical insights for a deeper understanding of tumors neuroimmune syndromes.

Case Characteristics and Diagnostic Challenges: Case 1 involved an elderly male presenting with acute unilateral limb weakness. He was initially suspected to have cerebrovascular disease. The later onset of painless vision loss, together with serum and cerebrospinal fluid AQP4 antibodies positive and longitudinally extensive spinal cord lesions, ultimately supported the diagnosis of NMOSD. This case underscores the importance of broadening differential diagnostic considerations in patients presenting with focal neurological deficits, particularly when disease progression extends beyond the typical manifestations of common cerebrovascular disorders. 

Case 2 highlights the diagnostic challenge of seronegative NMOSD. The patient was an elderly female presenting with symptoms of optic neuritis and myelitis. According to the 2015 IPND criteria, the initial cervical MRI finding of a focal intramedullary nodular lesion at the C5-C6 level was insufficient to diagnose AQP4-IgG-negative NMOSD. Therefore, we performed a systematic differential diagnosis: ① Intramedullary neoplasm (e.g., glioma): The lesion showed no significant enhancement and failed to account for the concomitant visual loss. ② Systemic rheumatic/autoimmune diseases (e.g., sarcoidosis, SLE): there was no evidence of systemic granulomatous disease, a negative rheumatologic antibody panel, and the clinical course did not support these diagnoses. ③ Other demyelinating diseases (e.g., MOG antibody-associated disease [MOGAD]): MOG-IgG was negative in both serum and CSF, the patient was elderly at onset, and she did not exhibit the characteristic brain MRI findings of MOGAD. ④ Infection (e.g., tuberculosis, viral myelitis): The patient had no fever or systemic infectious symptoms, and CSF analysis did not suggest infection. ⑤ Vascular disorders (e.g., spinal cord infarction, arteriovenous malformation): The clinical presentation lacked acute stroke-like features, the DWI hyperintensity was not accompanied by corresponding ADC restriction, and no flow voids were observed, thereby excluding a vascular etiology. Based on the above, and given that tumefactive or mass-like lesions mimicking neoplasm have been reported in NMO/NMOSD (12), the clinical suspicion of NMOSD was high even though the diagnosis was not confirmed at the initial presentation. Furthermore, CSF protein elevation was also consistent with acute demyelinating disease.

MRI obtained during the relapse revealed typical LETM extending from C1 to C3, with complete resolution of the original focal lesion. Concurrently, the patient again demonstrated a significant response to methylprednisolone pulse therapy. This clinical course fulfilled the 2015 IPND diagnostic criteria for AQP4-IgG-negative NMOSD (both optic neuritis and acute myelitis with LETM), thereby confirming the diagnosis of NMOSD. The value of this case resides in illustrating the evolution of a form of seronegative NMOSD that is frequently misdiagnosed at presentation — an isolated "pseudotumoral" demyelinating lesion — into a classic, diagnostic-specific LETM during longitudinal follow-up.

Clinical Significance of the Tumor Association: These two cases illustrate the heterogeneity of the temporal relationship between tumors and NMOSD. In Case 1, neurological symptoms appeared 1.5 years after gastric cardia cancer surgery. Following glucocorticoid therapy, MRI revealed suspicious vertebral metastatic lesions; however, as the patient was transferred to another hospital and subsequently lost to follow-up, it remains unclear whether tumor recurrence actually occurred. AQP4 immunohistochemistry on the patient's gastric cardia cancer tissue was negative. Although a paraneoplastic mechanism mediated by other antigens cannot be excluded, direct evidence supporting the classical molecular mimicry hypothesis is lacking. In Case 2, pancreatic cancer was diagnosed approximately two years after the initial onset of optic neuritis and myelitis. This relatively long interval precludes the exclusion of either a coincidental association or a shared susceptibility. Possible explanations include: the two diseases occurring together by chance in an elderly population; a common background of immune dysregulation that increases the risk of both autoimmune disease and cancer; long-term immunosuppressive therapy, which might theoretically affect immune surveillance, although its exact role in tumor development remains unclear. Consequently, a definitive paraneoplastic causal relationship between the tumor and NMOSD cannot be established in either case.

Treatment Response and Prognostic Considerations: Both patients exhibited a rapid and favorable clinical response to high-dose glucocorticoid pulse therapy, confirming the inflammatory nature of NMOSD lesions. Although the neurological symptoms of Case 1 improved, a follow-up MRI revealed suspicious metastatic lesions in the T9 and T11 vertebral bodies (the patient was transferred to another hospital and lost to follow-up without pathological confirmation, leaving the actual occurrence of tumor recurrence unclear). This finding suggests that vigilance for tumor recurrence or metastasis is necessary, but further evidence is needed to confirm this. The clinical course of Case 2 vividly illustrates how oncological outcomes ultimately determine neurological disease prognosis: despite repeated responsiveness of neurological relapses to glucocorticoid therapy, the patient ultimately succumbed to progression of pancreatic cancer. This underscores the necessity of a multidisciplinary approach in the management of NMOSD in patients with concurrent malignancies, aiming to strike a delicate balance between controlling neuroinflammation and administering antitumor therapy. 

Unresolved Issues: In Case 1, the nature of the suspicious lesions in the thoracic vertebrae (T9 and T11) remained indeterminate; the patient was transferred to an external institution and lost to follow-up. As no confirmatory diagnostic procedures, such as histopathological analysis or PET-CT, were conducted, whether tumor recurrence or metastasis occurred remains unclear. In Case 2, which represents seronegative NMOSD, AQP4 antibody retesting was indicated but not performed due to the patient's refusal after discussion. Furthermore, for such patients, the risk-benefit assessment of long-term immunosuppressive strategies lacks guidance from high-level evidence and necessitates highly individualized decision-making.

## Conclusion

The inclusion of patients with gastric cardia cancer and pancreatic cancer in this report expands the disease spectrum of tumor-associated NMOSD. The cases, characterized by stroke-like onset and pseudotumoral spinal lesions, highlight the critical role of serum AQP4-IgG testing and dynamic imaging follow-up in the diagnostic process. These findings suggest the importance of targeted malignancy evaluation, particularly in older patients, those with adenocarcinoma-associated presentations, atypical phenotypes, or when recurrence is suspected. Furthermore, they underscore the essential role of multidisciplinary collaboration in managing the complex interplay between oncological and neuroinflammatory disease processes.

## Data Availability

The original contributions presented in the study are included in the article/**Supplementary Material**. Further inquiries can be directed to the corresponding author.
