# Supplementary material for: Case Report: Neuromyelitis optica spectrum disorder associated with gastric cardia and pancreatic cancers: clinical features and oncological implications
Source: Front Oncol. 2026 Jun 3;16:1799562. doi: 10.3389/fonc.2026.1799562 (PMC13272177; doi:10.3389/fonc.2026.1799562)
Supplement: Supplementary file 1 [file DataSheet1.docx]

Supplementary Material

# Supplementary Figures and Tables

## Supplementary Figures


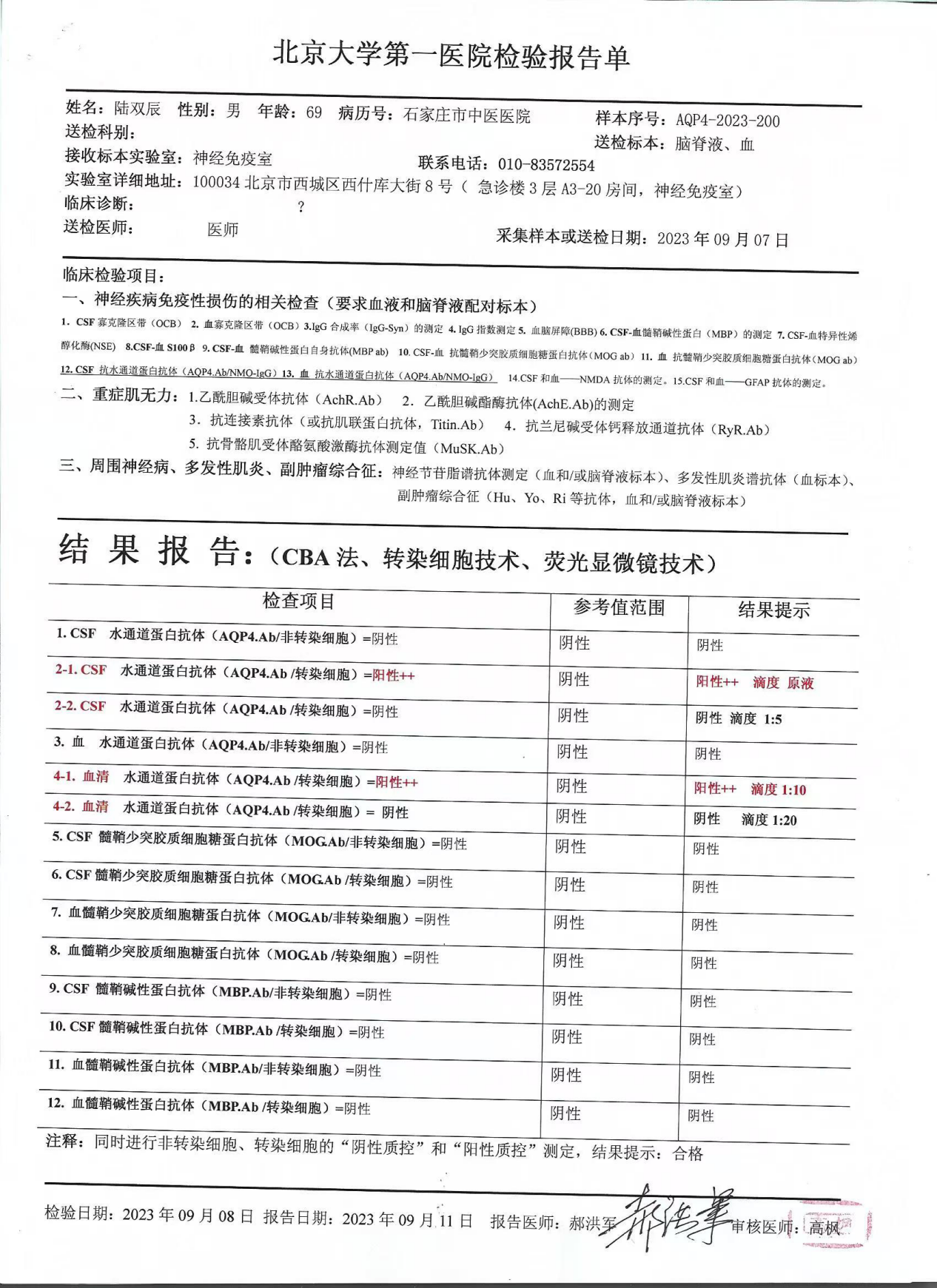


**Supplementary Figure 1**: Detection of AQP4‑Ab, MOG‑Ab, and MBP‑Ab in the serum and cerebrospinal fluid (CSF) of Case 1 using a cell‑based assay (CBA) at the Neuroimmunology Laboratory, Peking University First Hospital, with a report date of September 11, 2023. The results showed that CSF AQP4-Ab was strongly positive (titer: undiluted) and serum AQP4-Ab was strongly positive (titer: 1:10), whereas MOG-Ab and MBP-Ab tested negative.


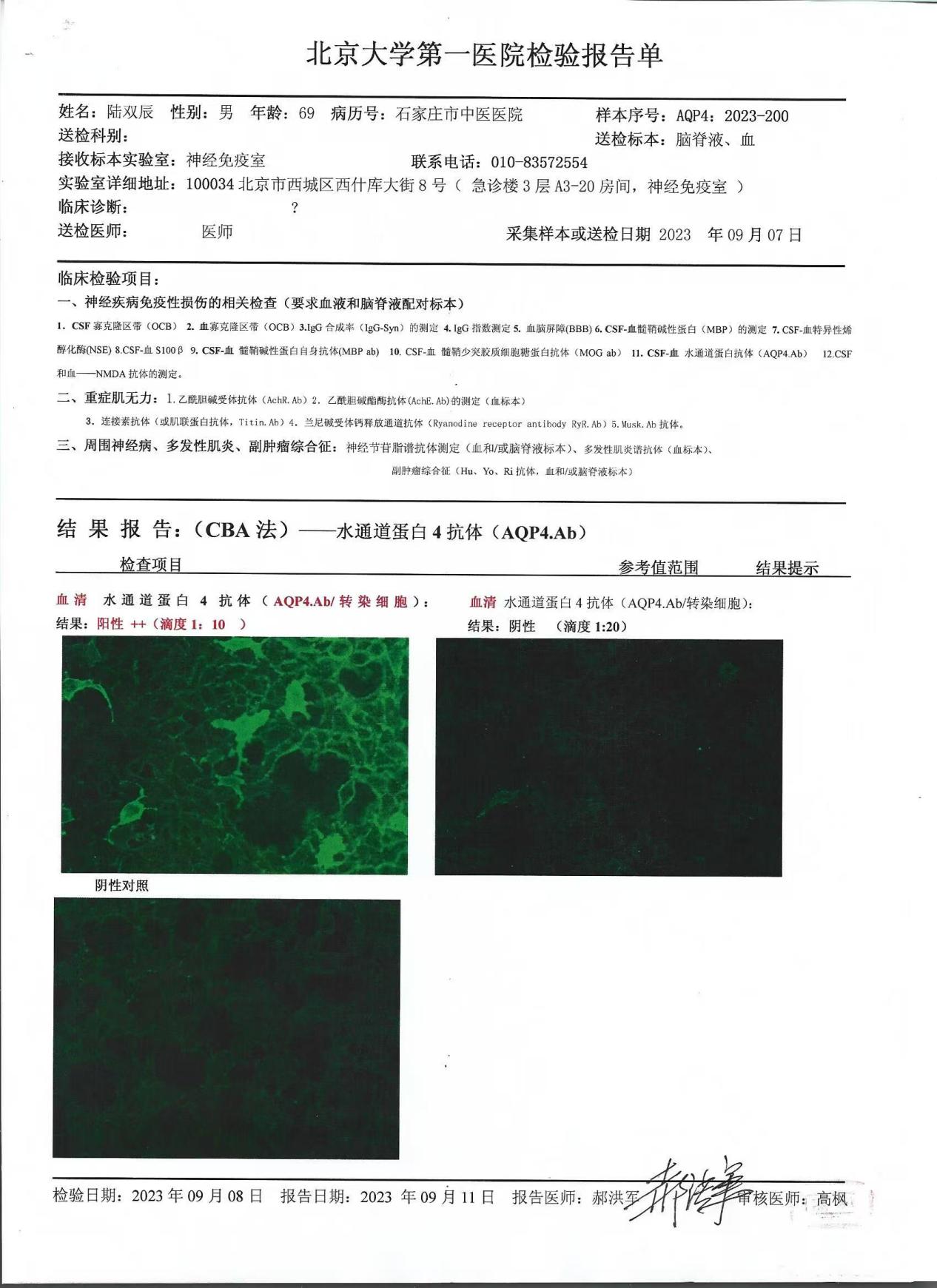


**Supplementary Figure 2.** Serum AQP4-Ab Detection of case 1. The result showed serum AQP4-Ab was strongly positive (titer: 1:10).


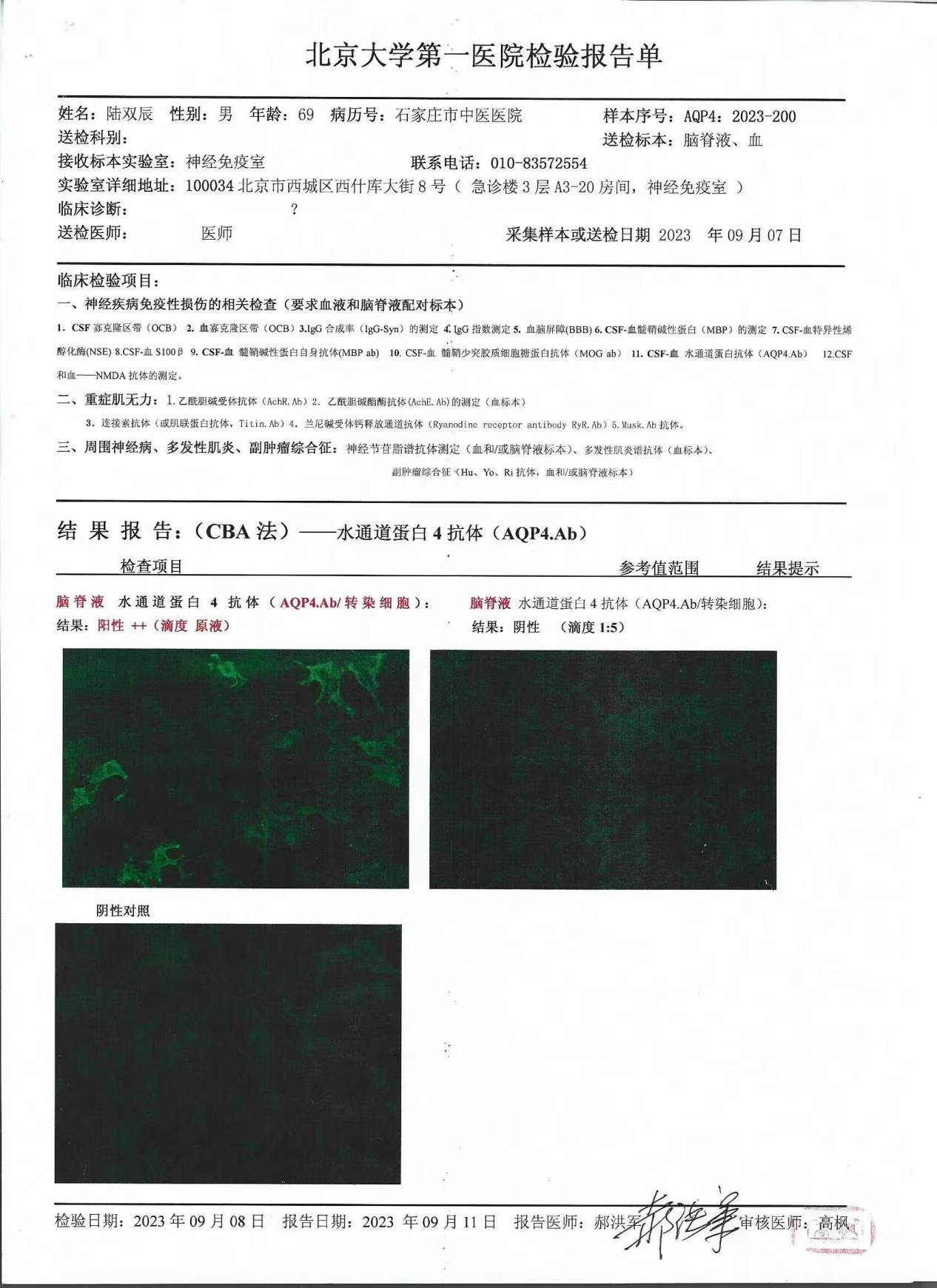


**Supplementary Figure 3.** Cerebrospinal Fluid (CSF) AQP4-Ab Detection of case 1. The result showed CSF AQP4-Ab was strongly positive (titer: undiluted).


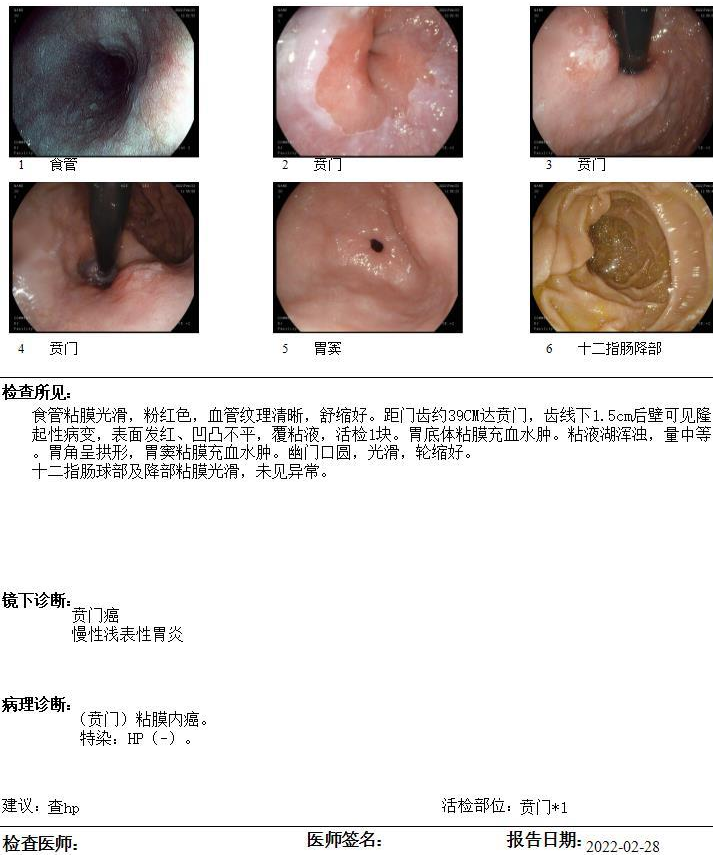


**Supplementary Figure 4.** Preoperative gastroscopy and pathological findings demonstrated gastric cardia cancer and chronic superficial gastritis.


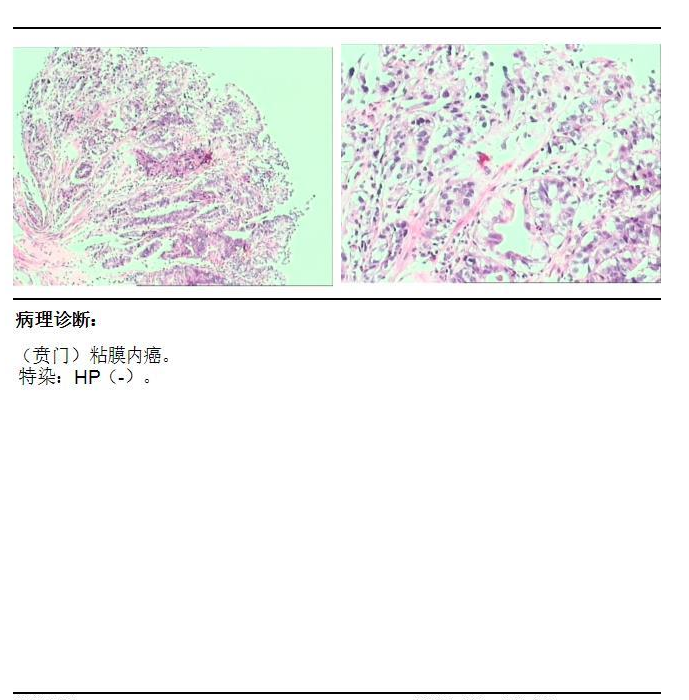


**Supplementary Figure 5.** Preoperative gastroscopy findings for Case 1 revealed intramucosal carcinoma located at the gastric cardia.


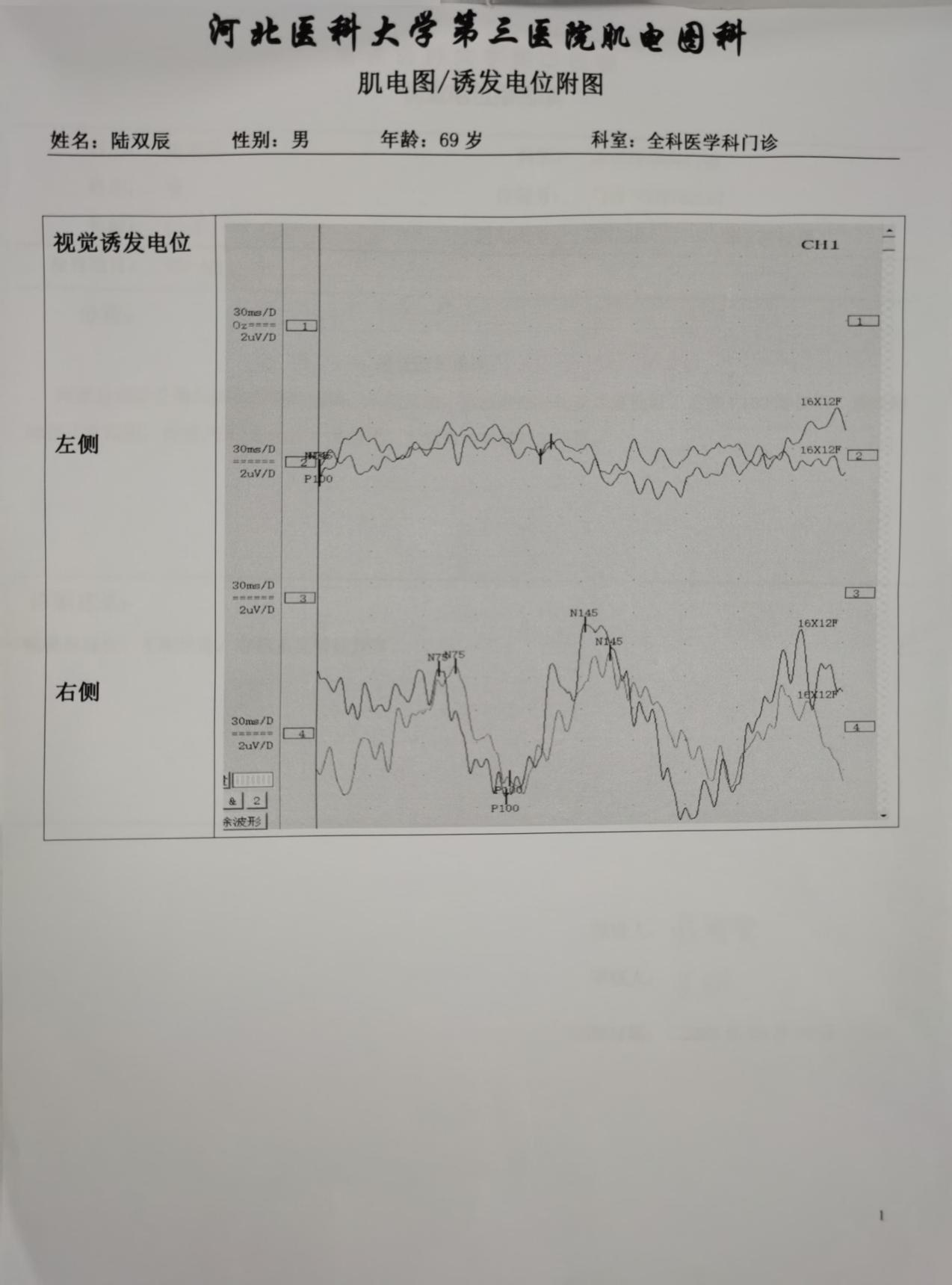


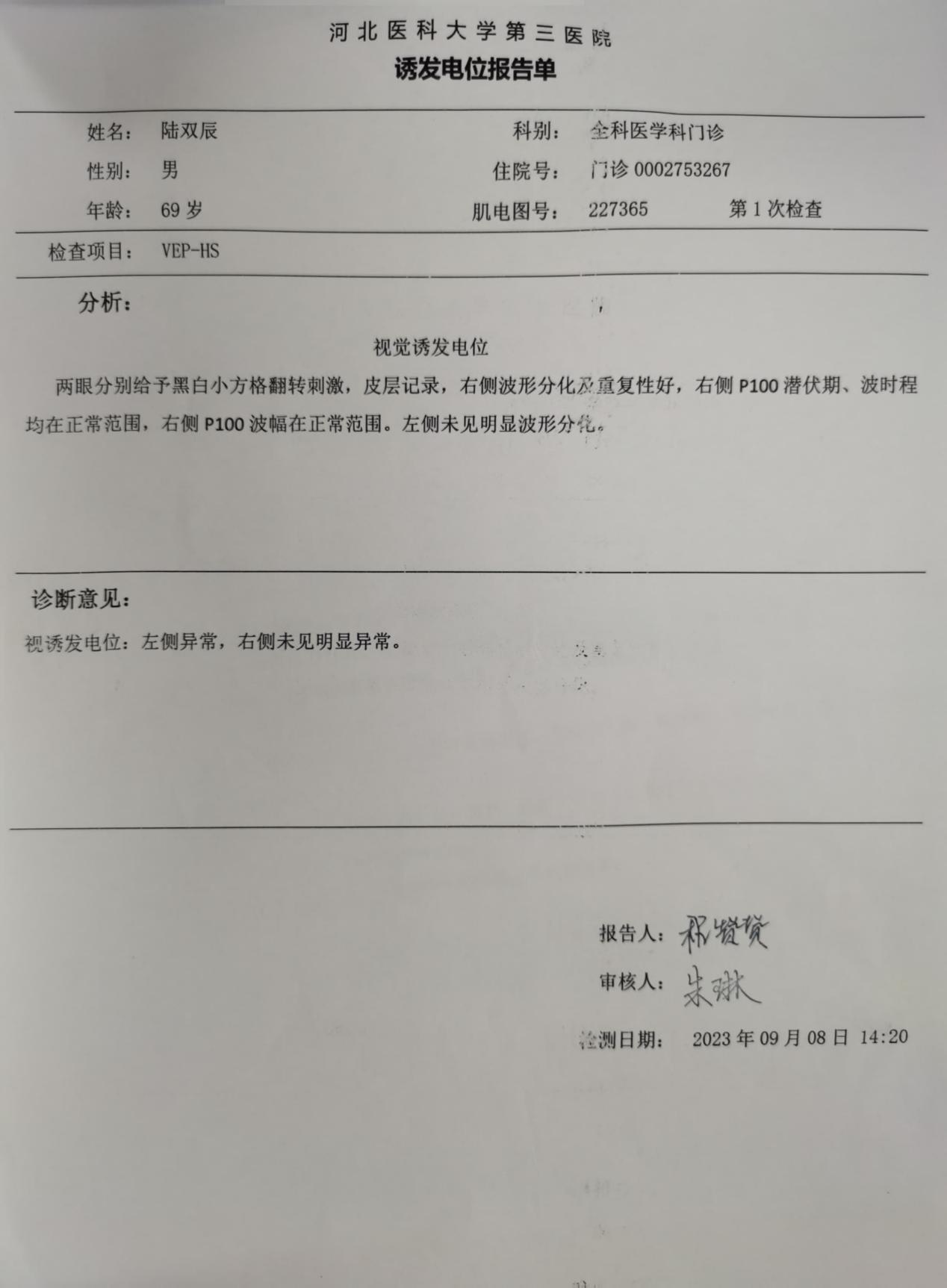


**Supplementary Figure 6.** The Visual Evoked Potentials (VEP) report for Case 1 showed an abnormality on the left side.


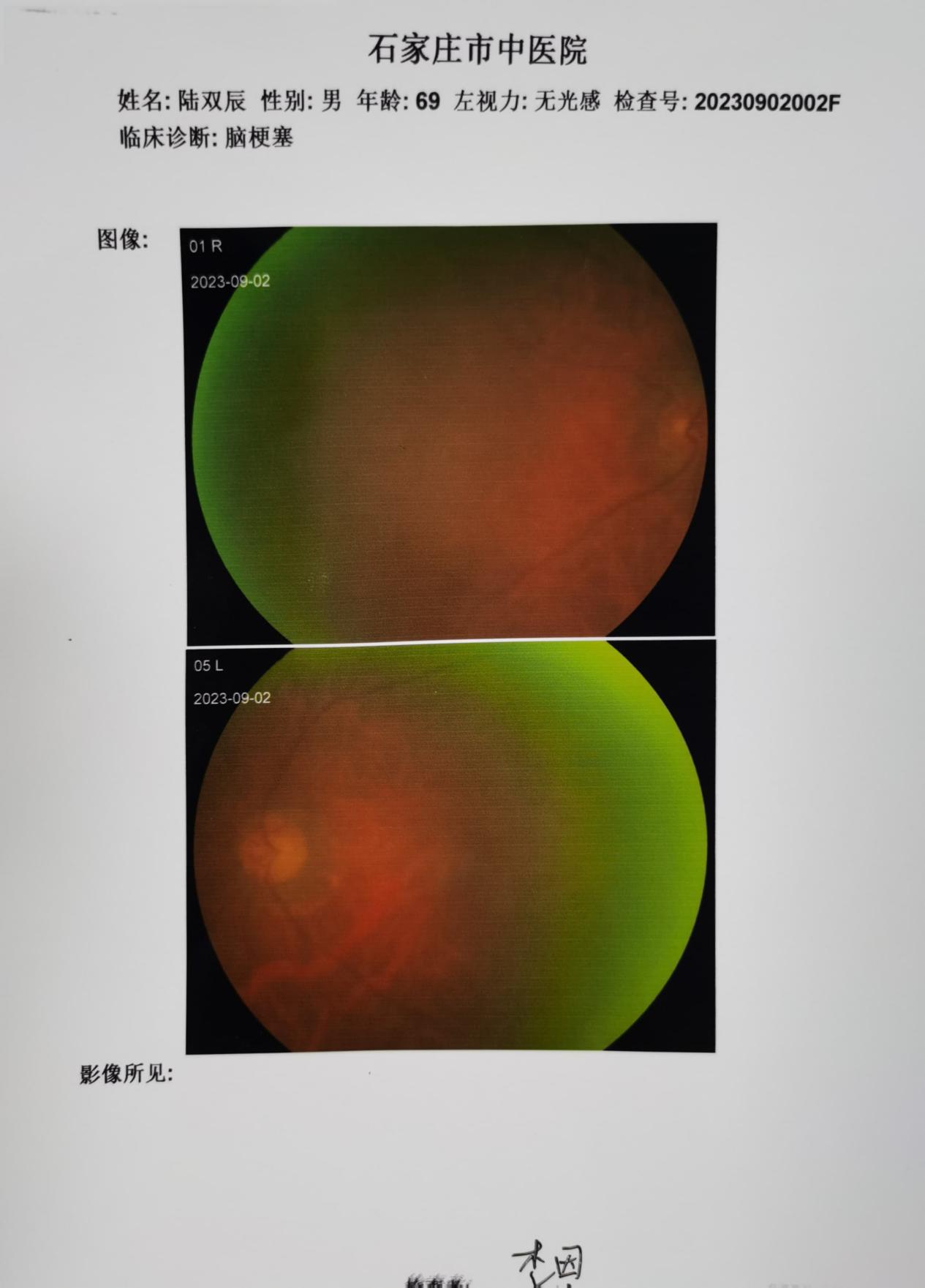


**Supplementary Figure 7.** Fundus photography in Case 1 revealed pallor of the left optic disc compared to the contralateral side.


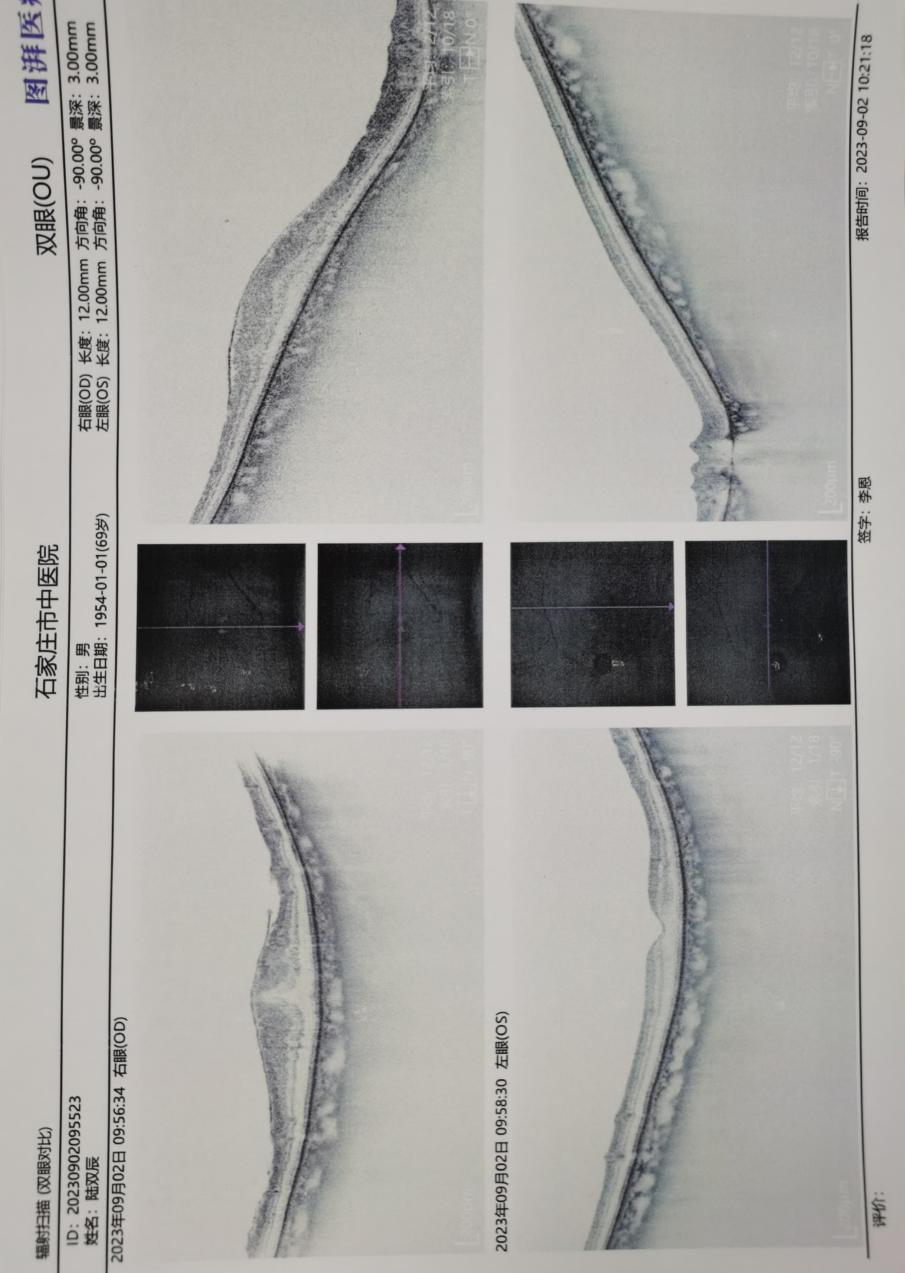


**Supplementary Figure 8.** Retinal optical coherence tomography (OCT) of Case 1 showed elevation in the macular area of the right eye with loss of normal contour and a hyperreflective band at the level of the nerve fiber layer.


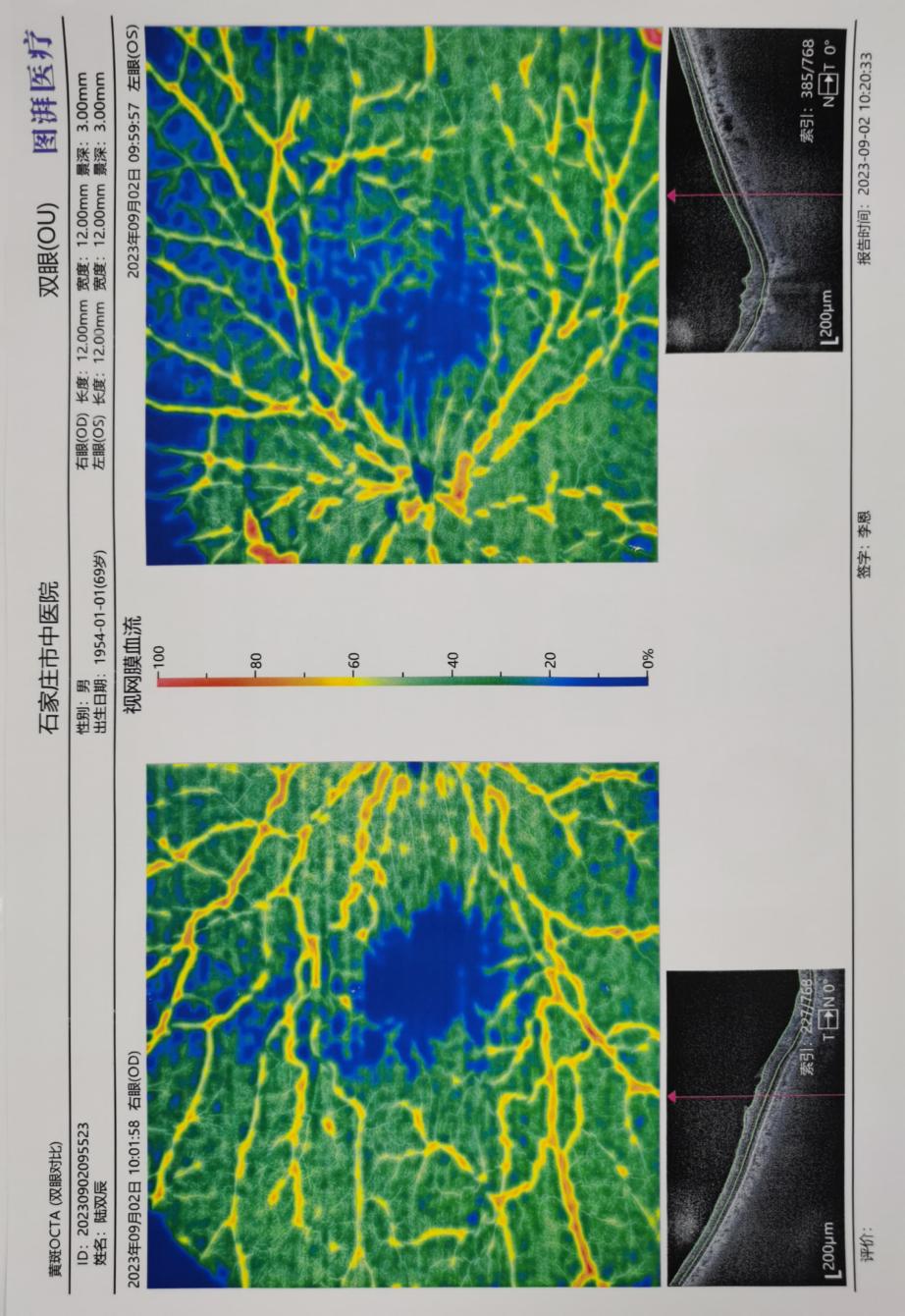


**Supplementary Figure 9.** Optical coherence tomography angiography (OCTA) in Case 1 demonstrated significantly reduced vessel density in the macular area of both eyes.


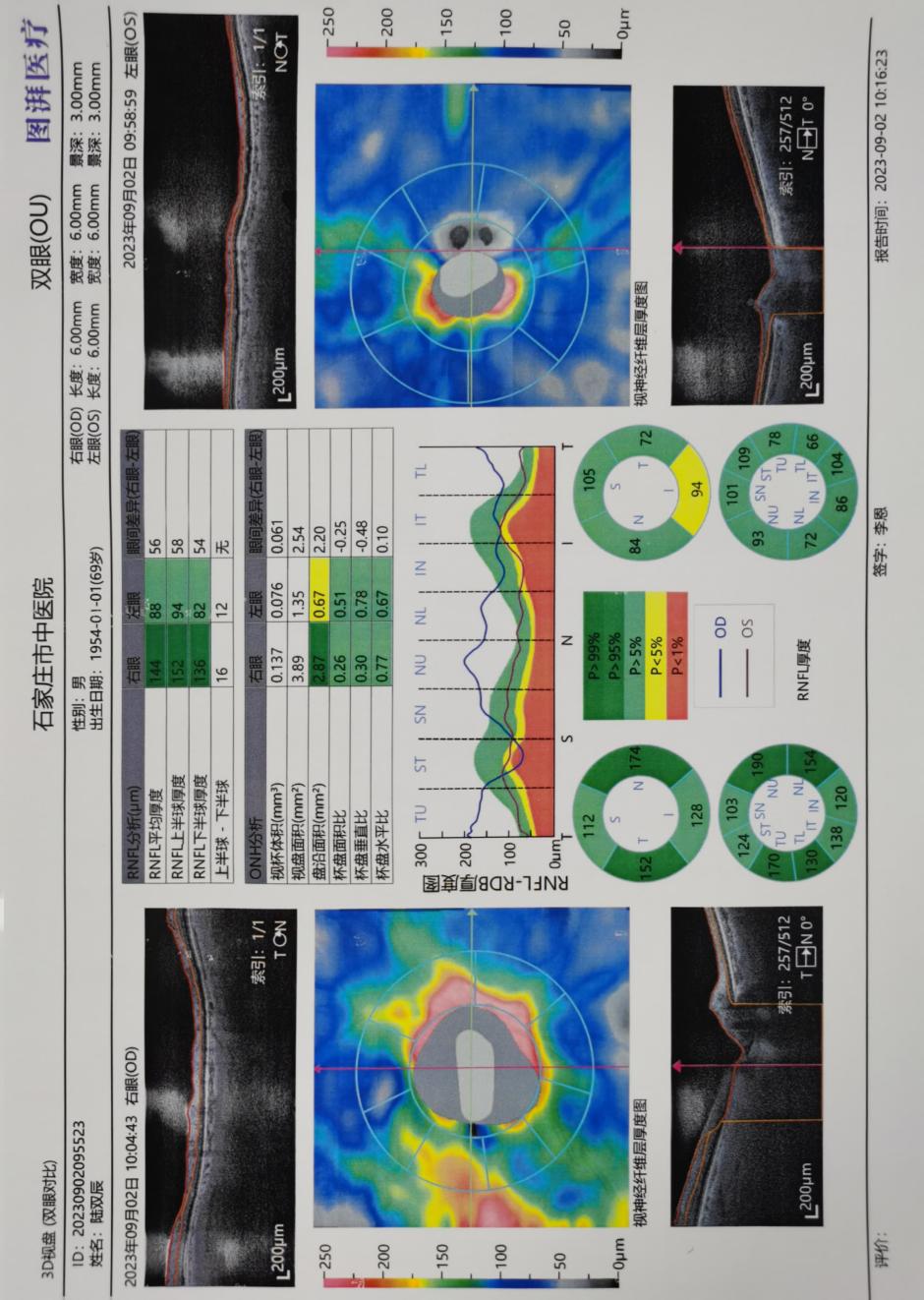


**Supplementary Figure 10.** Computerized image analysis of the retinal nerve fiber layer (RNFL) in Case 1 revealed borderline thickness of the fibers inferior to the left optic disc.


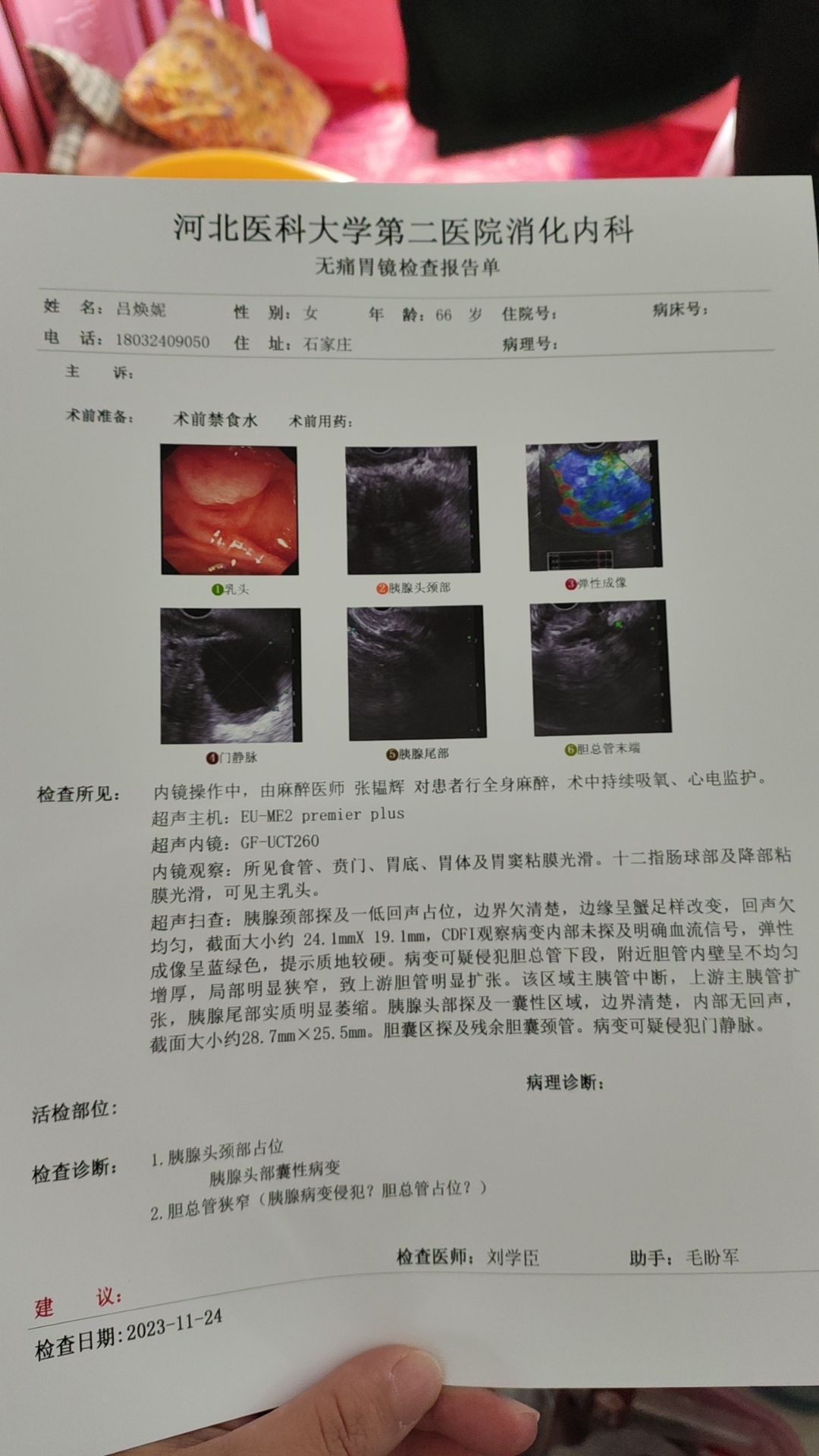


**Supplementary Figure 11.** In Case 2, the report of painless gastroscopy performed on November 24, 2023, showing: 1. A mass in the pancreatic head and neck, accompanied by a cystic lesion in the pancreatic head; 2. Common bile duct stricture (secondary to invasion by the pancreatic lesion versus a primary space-occupying lesion of the common bile duct).

**
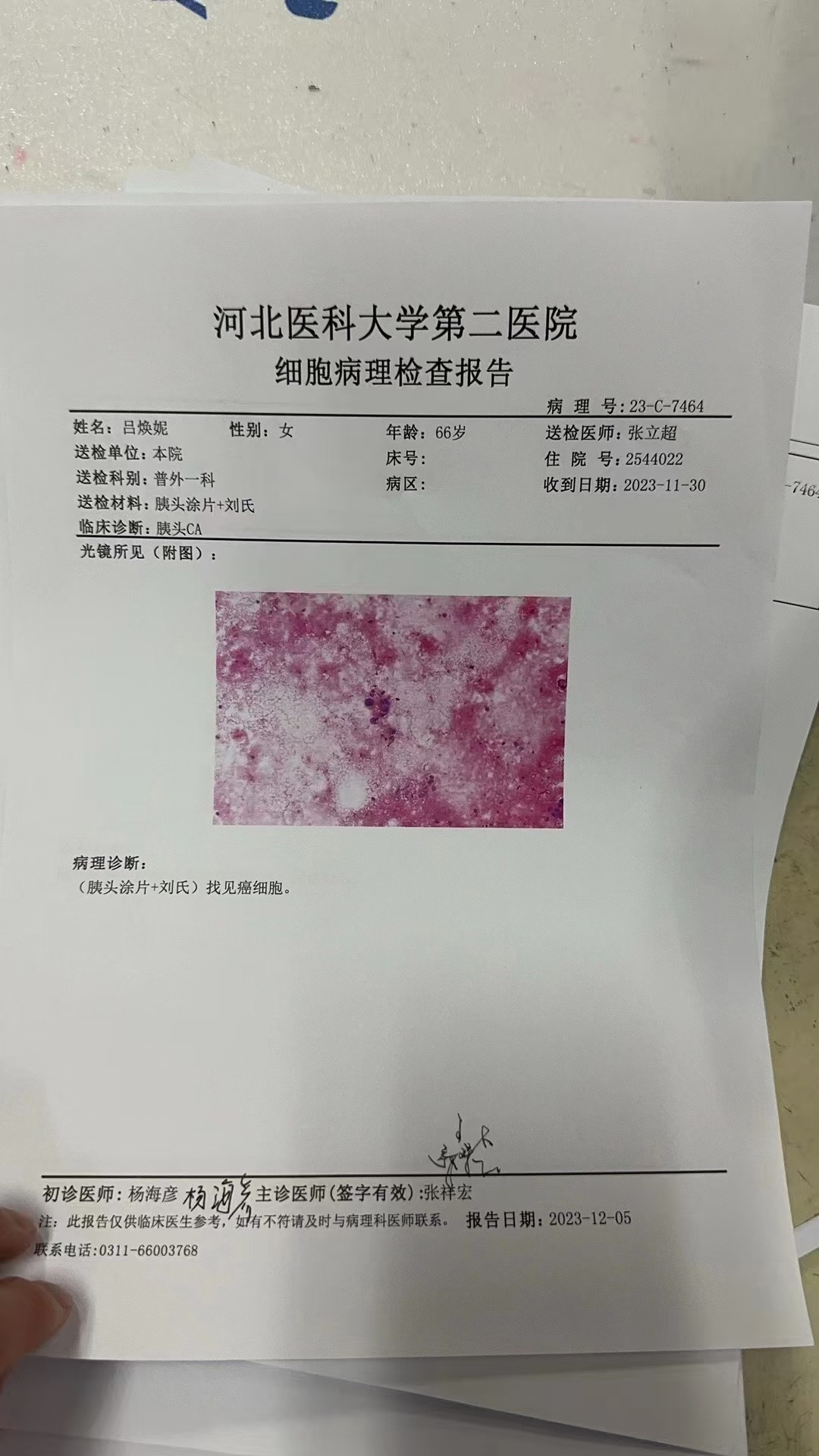
**

**Supplementary Figure 12.** Needle aspiration biopsy cytopathology report of Case 2 (submitted on November 30, 2023): (Pancreatic head smear and Liu's stain) – Cancer cells identified.

**
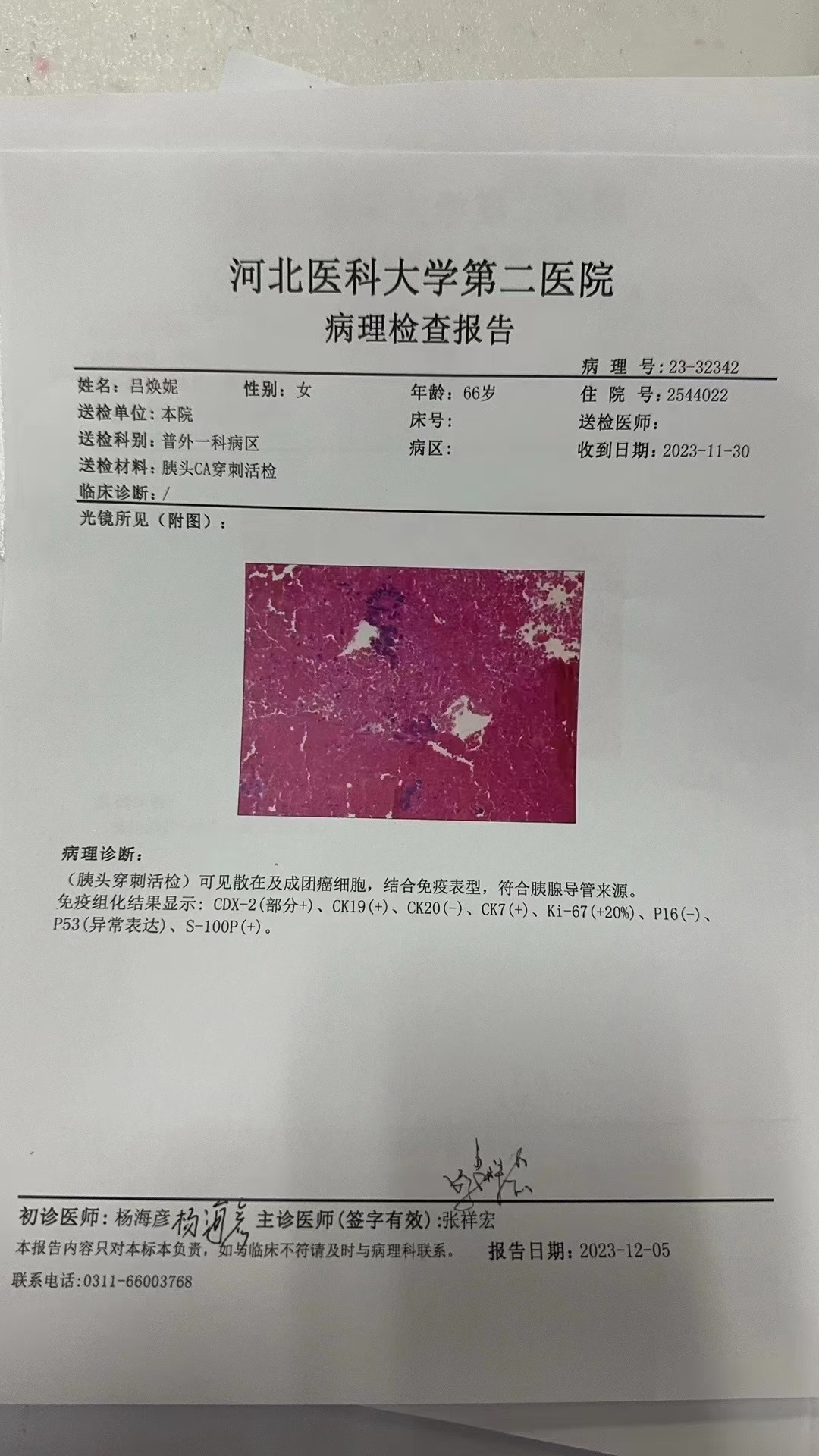
**

**Supplementary Figure 13.** In Case 2, pathology report of a pancreatic head needle biopsy revealing scattered and clustered malignant cells. When combined with the immunophenotypic profile, these findings are consistent with a pancreatic ductal origin. Immunohistochemistry results: CDX-2 (focal +), CK19 (+), CK20 (−), CK7 (+), Ki-67 (+20%), P16 (−), P53 (abnormal expression), S-100P (+).

# **
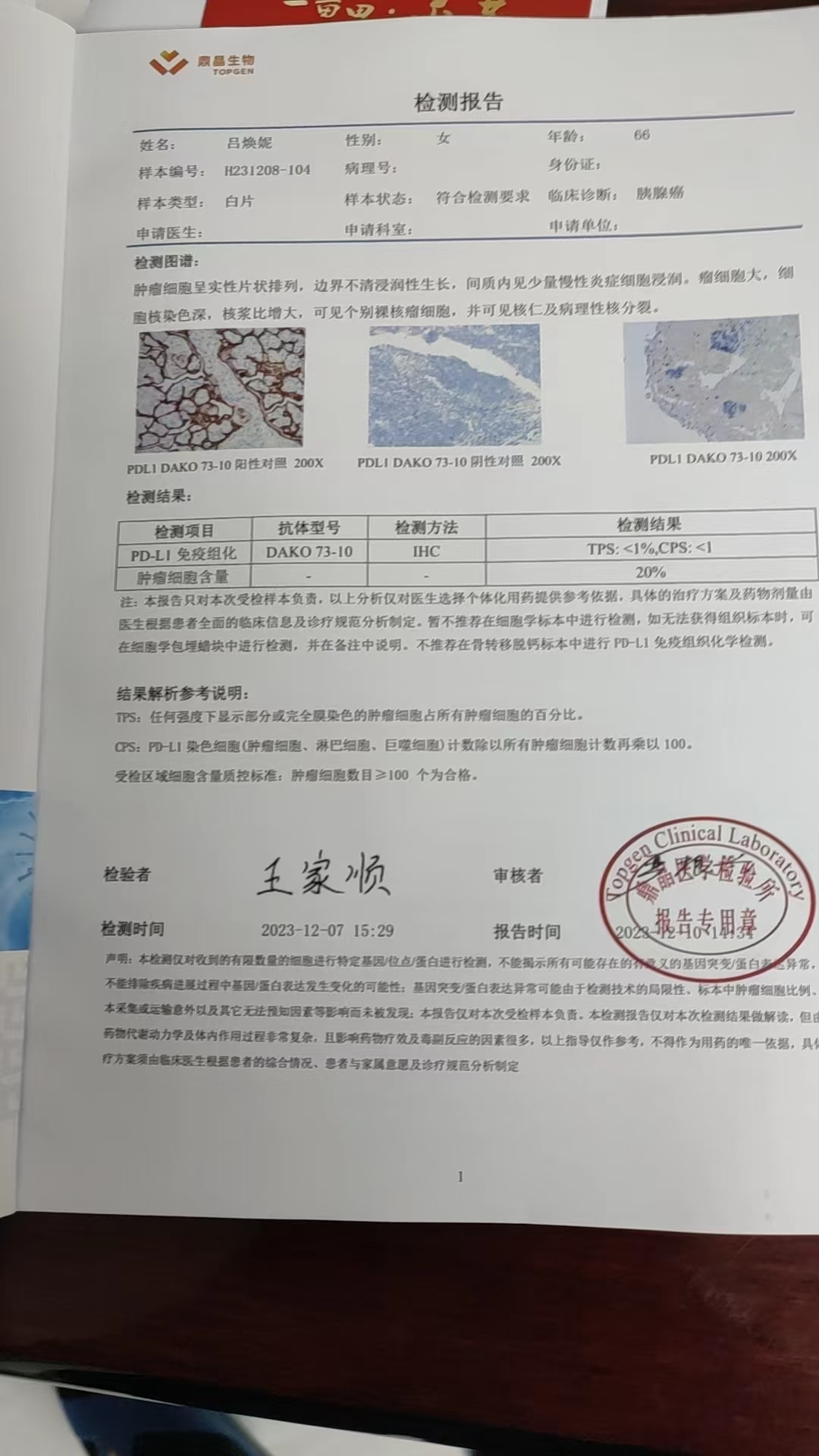
**

# **Supplementary Figure 14.** PD-L1 immunohistochemistry (IHC) results for Case2: TPS <1%, CPS <1, and tumor cellularity of 20%.

# 1.2 Supplementary Tables

**Supplementary Table 1**: Laboratory test results of Case 1

| Laboratory Test | Actual Result | Normal Range | **Report Date** |
| --- | --- | --- | --- |
| White Blood Cell Count (WBC) | 10.55*10^9^/L ↑ | 3.5-9.5*10^9^/L | 2023-08-25 |
| Percentage of neutrophils | 86.6% ↑ | 40.0-75.0% | 2023-08-25 |
| Percentage of lymphocytes | 9.3% ↓ | 20-50% | 2023-08-25 |
| Absolute value of neutrophils | 9.14*10^9^/L ↑ | 1.8-6.3*10^9^/L | 2023-08-25 |
| Absolute value of lymphocytes | 0.98*10^9^/L ↓ | 1.10-3.20*10^9^/L | 2023-08-25 |
| Red blood cell count（RBC） | 2.95*10^12^/L ↓ | 4.30-5.80*10^9^/L | 2023-08-25 |
| Hemoglobin | 88g/L ↓ | 130-175g/L | 2023-08-25 |
| Blood Urea | 6.90mmol/L | 3.60-9.50mmol/L | 2023-08-25 |
| Blood creatinine | 47.00umol/L ↓ | 57.00-111.00umol/L | 2023-08-25 |
| Total protein | 64.30g/L ↓ | 65.00-85.00g/L | 2023-08-25 |
| Albumin | 34.3g/L ↓ | 40.00-55.00g/L | 2023-08-25 |
| Alanine aminotransferase(ALT) | 17.10U/L | 9.00-50.00U/L | 2023-08-25 |
| Aspartate minotransferase(AST) | 18.70U/L | 15.00-40.00U/L | 2023-08-25 |
| d-dimer | 0.72ug/ml | 0.00-0.55ug/ml | 2023-08-25 |
| Carcinoembryonic antigen | 1.0ng/ml | 0.0-5.0ng/ml | 2023-08-25 |
| Alpha-fetoprotein | <1.3ng/ml | 0.0-7.0ng/ml | 2023-08-25 |
| Carbohydrate Antigen 125 | 8.5U/ml | 0.0-15.0U/ml | 2023-08-26 |
| Carbohydrate Antigen 19-9 | 20.2U/ml | 0.0-34.0U/ml | 2023-08-26 |
| Total prostate specific antigen | 1.8ng/ml | 0.0-4.0ng/ml | 2023-08-26 |
| Thyroid stimulating hormone（TSH） | 2.445mIU/L | 0.550-4.780mIU/L | 2023-08-26 |

**Supplementary Table 2**: Cerebrospinal fluid analysis of Case 1

| CSF Parameter | Actual Result | Normal Range | **Report Date** |
| --- | --- | --- | --- |
| Pressure | 150cmH_2_O | 80-180cmH_2_O | 2023-09-06 |
| Colour | Colorless | Colorless | 2023-09-06 |
| Appearance | Transparent | Transparent | 2023-09-06 |
| Coagulation | Not self-condensing | Not self-condensing | 2023-09-06 |
| Total number of nucleated cells | 4.00*10^6^/L | < 5 × 10^6^/L | 2023-09-06 |
| Protein Level | 0.67g/L | 0.15-0.45g/L | 2023-09-06 |
| Glucose Level | 3.13mmol/L | 2.50-4.40mmol/L | 2023-09-06 |
| Chlorine level | 118.1mmol/L ↑ | 99.0-110.0mmol/L | 2023-09-06 |

**Supplementary Table 3**: Laboratory test results of Case 2

| Laboratory Test | Actual Result | Normal Range | Report Date |
| --- | --- | --- | --- |
| White Blood Cell Count (WBC) | 6.30*10^9^/L | 3.5-9.5*10^9^/L | 2021-12-06 |
| Percentage of neutrophils | 74.80% | 40.0-75.0% | 2021-12-06 |
| Percentage of lymphocytes | 18.70% ↓ | 20-50% | 2021-12-06 |
| Absolute value of neutrophils | 4.71*10^9^/L | 1.8-6.3*10^9^/L | 2021-12-06 |
| Absolute value of lymphocytes | 1.18*10^9^/L | 1.10-3.20*10^9^/L | 2021-12-06 |
| Red blood cell count（RBC） | 4.14*10^12^/L | 3.8-5.1*10^9^/L | 2021-12-06 |
| Hemoglobin | 131.00g/L | 115-150g/L | 2021-12-06 |
| Blood Urea | 3.67mmol/L | 3.1-8.8mmol/L | 2021-12-06 |
| Blood creatinine | 59.00umol/L | 41-81.00umol/L | 2021-12-06 |
| Total protein | 73.40g/L | 65.00-85.00g/L | 2021-12-06 |
| Albumin | 43.30g/L | 40.00-55.00g/L | 2021-12-06 |
| Alanine aminotransferase(ALT) | 19.00U/L | 7.00-40.00U/L | 2021-12-06 |
| Aspartate minotransferase(AST) | 18.00U/L | 15.00-35.00U/L | 2021-12-06 |
| d-dimer | 0.45ug/ml | 0.00-0.50ug/ml | 2021-12-06 |
| Carcinoembryonic antigen | 0.6ng/ml | 0.0-5.0ng/ml | 2021-12-06 |
| Alpha-fetoprotein | 4.2ng/ml | 0.0-7.0ng/ml | 2021-12-06 |
| Carbohydrate Antigen 125 | 5.4U/ml | 0.0-16.0U/ml | 2021-12-07 |
| Carbohydrate Antigen 19-9 | 20.65U/ml | 0.0-34.0U/ml | 2021-12-07 |
| Carbohydrate Antigen 15-3 | 6.00ng/ml | 0.0-19U/ml | 2021-12-07 |
| Thyroid stimulating hormone（TSH） | 1.624mIU/L | 0.51-4.94mIU/L | 2021-12-07 |

**Supplementary Table 4**: Cerebrospinal fluid analysis of case 2

| CSF Parameter | Actual Result | Normal Range | Report Date |
| --- | --- | --- | --- |
| Colour | Pink and turbid* | Colorless | 2021-12-08 |
| Appearance | Transparent | Transparent | 2021-12-08 |
| Coagulation | Not self-condensing | Not self-condensing | 2021-12-08 |
| Red blood cell count | 4000.00*10^6^/L* ↑ | 0 | 2021-12-08 |
| Total number of nucleated cells | 1.00*10^6^/L | < 5 × 10^6^/L | 2021-12-08 |
| Protein Level | 1.50g/L ↑ | 0.15-0.45g/L | 2021-12-08 |
| Glucose Level | 3.19mmol/L | 2.50-4.50mmol/L | 2021-12-08 |
| Chlorine level | 129mmol/L | 123-130mmol/L | 2021-12-08 |

### *****This finding is attributed to a traumatic lumbar puncture, resulting in an elevated red blood cell count and a pinkish discoloration.

### **Supplementary Table 5.** Timeline for Case 1

| Time Point | Clinical Event |
| --- | --- |
| **1.5 years before admission** | Underwent radical surgery for gastric cardia cancer; pathology: intramucosal adenocarcinoma (pT1aN0M0, stage IA). Regular postoperative follow‑up. |
| **2 days before admission** | Sudden onset of right‑sided limb weakness. |
| **Day 0 (admission)** | Admitted to hospital. Physical exam: muscle strength grade IV in right limbs and left lower limb; bilateral Babinski sign positive. Head CT: lacunar infarct in left frontal deep region and leukodystrophy. Initial diagnosis: acute cerebral infarction. No significant improvement with treatment. |
| **Day 3 of hospitalization** | Sudden vision loss in left eye (no light perception). Ophthalmology: left pupil diameter ~5 mm, direct light reflex absent. OCT: decreased macular blood flow density bilaterally. Orbital CT: normal. VEP: abnormal on left. Brain MRI: punctate ischemic lesions in bilateral frontal and parietal lobes. Spine MRI: T2 hyperintense intramedullary lesion extending from C2 to C4 (Figure 1A). Additionally, focal rounded lesion in T11 vertebral body on T1‑weighted, T2‑weighted, and fat‑suppressed sequences. Serum and CSF AQP4‑IgG positive (cell‑based assay); MOG‑IgG and MBP‑IgG negative. **Diagnosis revised to NMOSD.** Received intravenous methylprednisolone pulse therapy. |
| **After treatment (same hospitalization)** | Left eye vision improved to counting fingers at 2 meters; right limb and left lower limb strength recovered to grade IV⁺. Discharged on oral methylprednisolone with gradual tapering. |
| **~1 month after discharge** | Developed significant low back pain. Follow‑up cervical MRI: improvement of spinal cord signal abnormality at C2–C4. Thoracolumbar MRI: T1 and T2 hypointense signals in T9 and T11 vertebral bodies, suspicious for metastatic lesions. |
| **Same time point** | Patient transferred to a higher‑level hospital and **lost to follow‑up**. |
| **Later** | Immunohistochemical staining for AQP4 on archived gastric cardia cancer tissue: **negative**. |

### **Supplementary Table 6.** Timeline for Case 2

| Time Point | Clinical Event |
| --- | --- |
| **>6 months before admission (approx. mid‑2021)** | Progressive visual impairment, numbness/weakness in both upper limbs, right upper limb muscle spasms and pain. |
| **December 2021** | Admitted to hospital. Physical exam: visual acuity OD 0.8, OS 0.6, no visual field defects; decreased pain sensation in both upper limbs, muscle strength grade IV. History: hypertension several years, laparoscopic cholecystectomy 8 years prior. Cervical MRI: intramedullary nodular lesion at C5–C6 (T2/DWI hyperintense, no enhancement). CSF protein 1.5 g/L. Serum/CSF AQP4‑IgG, MOG‑IgG, MBP‑IgG negative (CBA). ENA, tumor markers, rheumatology panels negative. **Strongly suspected NMOSD.** Received methylprednisolone pulse therapy. |
| **After treatment (same hospitalization)** | Visual acuity improved; upper limb numbness/weakness reduced; muscle spasms/pain relieved. Muscle strength recovered to grade V⁻. Discharged on oral prednisone 5 mg daily maintenance. |
| **November 16, 2023** | Presented with painless progressive jaundice. |
| **November 17, 2023** | Abdominal MRI: cystic lesion in pancreatic head; mild dilation of hilar bile duct and common bile duct; small perisplenic fluid; gallbladder absent. |
| **November 27, 2023** | Contrast‑enhanced CT: lesions in pancreatic head/body/tail with cystic lesion in neck (suspicious for neoplasm); diffuse abnormal enhancement of common bile duct pancreatic segment causing biliary obstruction; heterogeneous decreased liver density (suggestive of hepatic injury); gallbladder not visualized. |
| **November 29, 2023** | Underwent EUS‑FNA, ERCP, endoscopic sphincterotomy, and biliary stent placement. Pathological examination: scattered and clustered cancer cells, consistent with pancreatic ductal origin. **Diagnosis: pancreatic cancer.** Prednisone discontinued (possible surgery planned). Switched to mycophenolate mofetil but stopped due to intolerance. |
| **December 13, 2023** | Started chemotherapy + immunotherapy: nab‑paclitaxel + gemcitabine (IV). |
| **December 14, 2023** | Received sintilimab (IV). |
| **December 20, 2023** | Second infusion of nab‑paclitaxel + gemcitabine. |
| **December 2023 – April 2024** | Regimen repeated ~every 2 weeks for 6 cycles. |
| **April 30, 2024** | Last cycle of chemotherapy completed. |
| **July 2024** | Relapse of NMOSD: numbness, weakness, muscle spasms in upper limbs. Cervical MRI: longitudinally extensive T2 hyperintense signals from C1 to C3; prior C5–C6 lesion resolved. Received repeat methylprednisolone pulse therapy → significant improvement. |
| **August 2024** | Follow‑up MRI: marked reduction of abnormal signals. Pancreatic cancer continued to progress. |
| **Later (date not specified)** | Developed pyloric obstruction, required jejunostomy. |
| **March 2025** | Patient died from tumor progression. |
